# Soft skin-interfaced mechano-acoustic sensors for real-time monitoring and patient feedback on respiratory and swallowing biomechanics

**DOI:** 10.1038/s41746-022-00691-w

**Published:** 2022-09-20

**Authors:** Youn J. Kang, Hany M. Arafa, Jae-Young Yoo, Cagla Kantarcigil, Jin-Tae Kim, Hyoyoung Jeong, Seonggwang Yoo, Seyong Oh, Joohee Kim, Changsheng Wu, Andreas Tzavelis, Yunyun Wu, Kyeongha Kwon, Joshua Winograd, Shuai Xu, Bonnie Martin-Harris, John A. Rogers

**Affiliations:** 1 Querrey-Simpson Institute for Bioelectronics, Northwestern University, Evanston, IL USA; 2 Department of Ocean System Engineering, Jeju National University, Jeju, Republic of Korea; 3 Department of Biomedical Engineering, Northwestern University, Evanston, IL USA; 4 Department of Communication Sciences and Disorders, Northwestern University, Evanston, IL USA; 5 Department of Materials Science and Engineering, National University of Singapore, Singapore, 117575 Singapore; 6 Institute for Health Innovation and Technology, National University of Singapore, Singapore, 117599 Singapore; 7 School of Electrical Engineering, Korea Advanced Institute of Science and Technology, Daejeon, Republic of Korea; 8 Department of Materials Science and Engineering, Northwestern University, Evanston, IL USA; 9 Department of Dermatology, Northwestern University Feinberg School of Medicine, Chicago, IL USA; 10 Department of Otolaryngology-Head and Neck Surgery and Radiation Oncology, Northwestern University Feinberg School of Medicine, Chicago, IL USA

**Keywords:** Biomedical engineering, Quality of life

## Abstract

Swallowing is a complex neuromuscular activity regulated by the autonomic nervous system. Millions of adults suffer from dysphagia (impaired or difficulty swallowing), including patients with neurological disorders, head and neck cancer, gastrointestinal diseases, and respiratory disorders. Therapeutic treatments for dysphagia include interventions by speech-language pathologists designed to improve the physiology of the swallowing mechanism by training patients to initiate swallows with sufficient frequency and during the expiratory phase of the breathing cycle. These therapeutic treatments require bulky, expensive equipment to synchronously record swallows and respirations, confined to use in clinical settings. This paper introduces a wireless, wearable technology that enables continuous, mechanoacoustic tracking of respiratory activities and swallows through movements and vibratory processes monitored at the skin surface. Validation studies in healthy adults (*n* = 67) and patients with dysphagia (*n* = 4) establish measurement equivalency to existing clinical standard equipment. Additional studies using a differential mode of operation reveal similar performance even during routine daily activities and vigorous exercise. A graphical user interface with real-time data analytics and a separate, optional wireless module support both visual and haptic forms of feedback to facilitate the treatment of patients with dysphagia.

## Introduction

Swallowing, a primary function of human survival, is a highly complex synergy of rapid and interdependent movements triggered through sensory end organs in the oral cavity, pharynx, and larynx that not only serve to protect the airway from aspiration but also propel ingested material (bolus) throughout the upper aerodigestive tract^1–3^. The significance of swallowing function is highlighted by the millions of adults who suffer from swallowing problems (dysphagia) related to neurologic conditions, head and neck cancer, and gastrointestinal and respiratory diseases^4^. Diseases or conditions that impair central nervous system mechanisms that control swallowing or directly affect peripheral structures involved in swallowing movements can result in swallowing difficulty (dysphagia). Swallowing disorders range from mild to life-threatening, often with life-altering problems that can present significant rehabilitation challenges^3,5,6^. Dysphagia may involve altered sensation that delays the onset of swallowing movements, reductions in muscle strength, and decreased range and coordination of the timing of movements^7–10^. Adding to the complexity of the control and execution of swallowing, breathing must be intimately timed with swallowing initiation because the pharyngeal cavity, common to both functions, interchanges between patency during breathing to facilitate effortless movement of air to tight compression during swallowing, generating high positive pressures on the bolus required for clearance through the upper aerodigestive tract^7,11^.

Respiratory-swallowing coordination is critical for safely and efficiently transporting foods and liquids from the mouth into the esophagus. In healthy adults, the timing of swallow initiation typically corresponds with a pause in the expiratory phase of quiet breathing at mid-to-low lung volumes^7,8,10–12^. This coordinative pattern (1) serves as a vital mechanism for airway protection, (2) facilitates physiological events beneficial to swallowing safety and efficiency, such as tongue base retraction, laryngeal elevation, and pharyngoesophageal segment opening, and (3) subsequently aids in bolus clearance^13–15^. However, it is well-documented that the coordination of breathing with swallowing is significantly disrupted in patients with dysphagia, resulting in impairments in the swallowing mechanism and significant decreases in health and quality of life. Traditional swallowing interventions typically use a single-system approach, focusing on increasing the strength and range of motion of oral, pharyngeal, and laryngeal structures^1,16^. Current methods enable detection of swallowing events and respiratory phase with piezoresistive/surface electromyography sensors mounted on the neck, but they rely on wired hardware that cannot be easily adapted for use in home settings or during natural daily activities without patient burden^17^. As such, despite demonstrated positive outcomes of these schemes, most patients continue living with swallowing impairments. More recently, a novel intervention that trains patients with dysphagia to initiate swallowing during the expiratory phase of the breathing cycle has shown to decrease aspiration and improve swallowing biomechanics in some patient populations^18^. However, three significant methodologic challenges exist in the rehabilitation of swallowing function: (1) the ability to unambiguously detect the occurrence of swallowing and swallowing coordinated with breathing without the use of expensive, non-portable imaging and respiratory recording equipment; (2) the ability to ensure the fidelity of the swallowing intervention and provide visual cueing to enhance performance in real-time, and (3) the ability to facilitate stability of the acquired swallowing skills through ambulatory monitoring and cueing. The work presented here addresses these challenges with a device technology that captures subtle and gross motions at the surface of the skin using soft, miniaturized wireless sensors mounted near the base of the neck.

The basic principles of such types of mechano-acoustic (MA) measurements of body processes can be found elsewhere^19–22^. Briefly, a wide-bandwidth inertial measurement unit (IMU) captures motions at the skin’s surface, ranging from vibratory oscillations associated with vocalizations and cardiac sounds to bulk body movements related to walking and jumping. The resulting data reflect diverse streams of information that can be analyzed using digital filtering and machine learning techniques^23–28^. MA devices that use multiple IMUs for differential detection offer enhanced capabilities in monitoring physiological parameters, particularly those that arise from cardiopulmonary activity, even in scenarios that involve significant motion-related artifacts, as described recently^29–33^.

This paper presents an MA sensor specifically designed for real-time monitoring of swallowing and breathing. The system exploits dual IMUs arranged in a differential configuration and interfaced to the neck and upper chest to optimize the strength of the relevant components of the signal and to eliminate confounding influences of motion artifacts. The results include a fully wireless system and an associated set of analysis algorithms capable of continuously detecting swallowing events and the surrounding respiratory phase patterns across subjects with different body types and sizes. Validation studies that examine respiratory-swallow phase patterning and swallow detection in healthy adults (*n* = 67) and patients with dysphagia (*n* = 4) reveal performance comparable to or better than clinical standard equipment in controlled settings. The dual measurement approach also allows similar measurement fidelity during natural daily activities. An optional wireless module for haptic feedback and a graphical user interface with real-time data analytics provide continuous visual and tactile cues for swallow initiation. These advances have strong potential as the basis for practical technologies to monitor swallowing function in patients with dysphagia and to augment traditional rehabilitation approaches.

## Results and discussion

### Skin-integrated wireless device for monitoring swallowing and respiratory activity

The results presented here focus on adapting this technology and optimizing it for monitoring swallowing (SW) events and their timing relative to respiratory phases (i.e., inspiration, expiration). The devices address the rehabilitation needs of millions of adults who suffer from swallowing problems (dysphagia) related to neurologic conditions, head and neck cancer, and gastrointestinal and respiratory diseases. The architecture exploits an umbilical design that positions one IMU in mechanical communication with the throat to capture swallows and a second IMU with the upper chest to capture respirations. For most patients, the former location is the suprasternal notch (SN), and the latter is the sternal manubrium (SM). In individuals whose swallows induce only small amplitude motions of the throat, the former can be located above the SN, up to and including locations coincident with the laryngeal prominence (LP), to increase the magnitude of the signal. Differential measurements based on data from these two IMUs eliminate common mode features associated with physical activity.

**Fig. 1:**
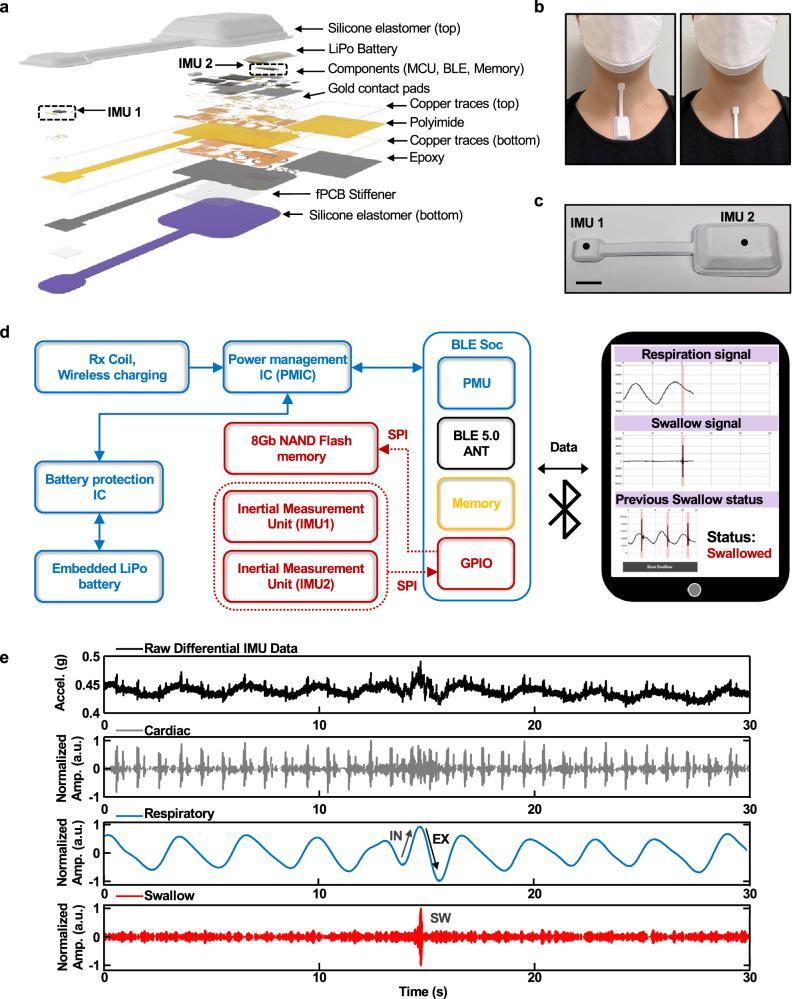
Device layout, operating principles, user interface, and representative measurements. **a** Exploded view schematic illustration of the umbilical MA device. **b** Image of the device attached on the LP(IMU1)/SN(IMU2) (left) and SN(IMU1)/SM(IMU2) (right) to capture respiratory activity and swallowing events. **c** Image of a device with a scale bar and illustration of the locations of IMU1 and IMU2 (scale bar = 1 cm). **d** System block diagram of device operation and illustration of a graphical user interface with real-time data analytics. **e** Representative differential data from IMU1/IMU2. Graphs from top to bottom show data: unprocessed; bandpass filtered between 15 and 60 Hz to highlight features associated with cardiac cycles; bandpass filtered between 0.1 and 0.8 Hz to highlight respiratory cycles; and high pass filtered at 90 Hz to highlight features related to swallowing.

Figure 1a presents an exploded view illustration of this umbilical mechano-acoustic (UMA) device, highlighting the two IMUs and the umbilical connection (length, 7 cm), the soft encapsulating structure that seals the electronic components, and the segmented design of the flexible printed circuit board (fPCB) that allows folding and stretching as part of the packaging and skin-interfaced mounting aspects of the system, respectively. The folded design reduces the size of the device to eliminate bending in regions of the fPCB that support the microcontroller and other related integrated circuit components to avoid mechanical failures at the solder joints. The combined use of low-modulus elastomeric structural materials (0.3 mm thickness silicone encapsulation; Silbione RTV 4420), serpentine architectures in the fPCB, and commercial off-the-shelf components yields a manufacturable, cost-effective platform that can mount on the skin with a conventional thin silicone acrylate double-sided biomedical adhesive. This compliant interface avoids irritation or discomfort even in the sensitive regions of the SN, SM, and LP. Figure 1b shows the device mounted on the base of the neck. Figure 1c highlights the umbilical design, configured to minimize interference related to neck movements. The dimensions of the main body of the encapsulated device are 34 mm by 22 mm, and the weight is 7.4 g.

The block diagram in Fig. 1d summarizes the critical components of the device: (1) power management electronics and a rechargeable lithium-polymer (Li-Po) battery (75 mAh) to supply power to all components, (2) near-field communication (NFC) charging components and rectifiers to enable wireless charging, with overvoltage/overcurrent protection and thermal management, (3) dual high bandwidth IMUs to acquire MA signals via an integrated triaxial accelerometer (200 Hz sampling rate for *x* and *y* axes and 1600 Hz sampling rate for *z*-axis accelerations) through digital serial peripheral interface (SPI) communication, (4) 8-gigabit NAND flash memory to store acquired accelerometry data, (5) Bluetooth low energy (5.0) system on a chip (BLE SoC) microcontroller with a Bluetooth radio to enable wireless data collection and high-speed data acquisition, and (6) associated passive components. A custom iPad application designed for Apple’s iOS (iPad, Fig. 1d) serves as a graphical user interface to record, store, and display swallows and respiratory phase data in real-time. Figure 1e shows representative data and digitally filtered representations highlighting cardiac activity, respiratory cycles, and swallow events. The device leverages commercial off-the-shelf components assembled in an integrated package that is soft, small, and lightweight. This system effectively couples the IMUs to two different locations along the neck and upper chest to allow for continuous recording of respiratory activity and swallowing events, designed specifically for patients with dysphagia.

### Validation studies

Measurements of swallowing events and surrounding respiratory phase patterns provide significant clinical insights related to dysphagia diagnosis and treatment. Healthy adults typically initiate swallows during the expiratory phase of the breathing cycle at mid-to-low lung volumes^7,11^. The respiratory phase in which swallowing is initiated influences the biomechanics necessary for airway protection and efficient clearance of food and liquid (bolus) through the pharynx. As a result, monitoring respiratory-swallow coordination and training the initiation of swallows during the expiratory phase of breathing in patients with dysphagia have been shown to lead to improved airway protection and pharyngeal clearance^8,34^. In research settings, the current gold-standard method for assessing swallowing events and respiratory-swallow patterning includes two techniques: (1) Respiratory inductance plethysmography (RIP), which measures the overall expansion of the ribcage (RC) and abdomen (AB) via inductance coils, and (2) nasal cannulas that monitor pressure differentials at the nasal cavity as an absolute measurement of nasal airflow. Capturing synchronous nasal airflow and RC/AB kinematics allows for an accurate assessment of respiratory-swallow phase patterning.

**Fig. 2:**
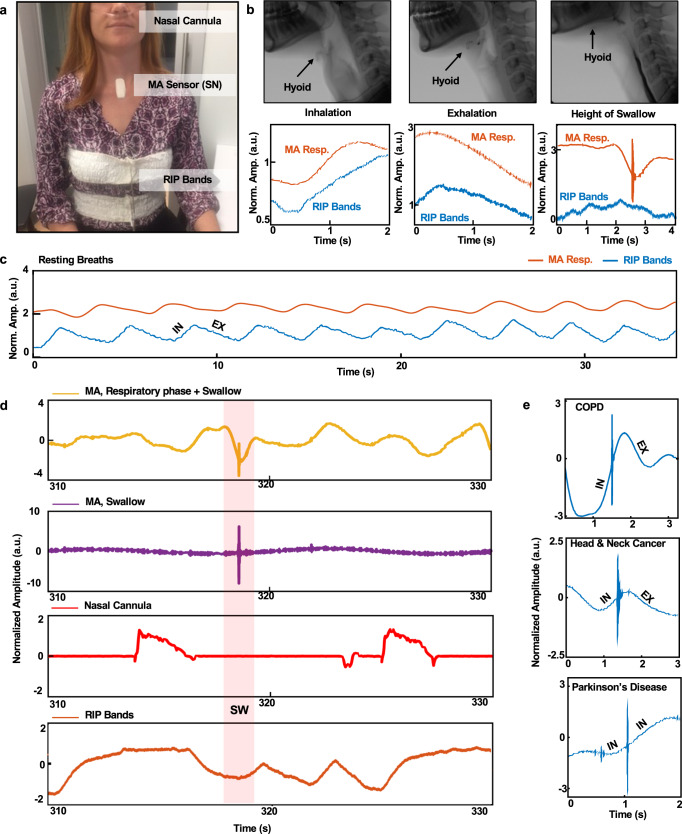
Simultaneous measurements of swallowing and respiratory cycles. **a** Picture of a healthy subject wearing a mechano-acoustic (MA) device at the SN, a nasal cannula, and respiratory inductance plethysmography (RIP) bands. **b** Images associated with a modified barium swallow study (MBSS) of a patient suffering from dysphagia and corresponding MA measurements. **c** Respiratory phase during rest breaths performed by a healthy subject. **d** Sample swallow event from a healthy subject with synchronized combined MA respiratory phase and swallow signal (yellow), filtered MA swallow signal (purple), nasal airflow signal (red), and calculated RIP signal (orange). **e** Various swallows from chronic obstructive pulmonary disease (COPD), head and neck cancer, and Parkinson’s disease patients, highlighting the importance of swallow training for patients suffering from dysphagia.

Validation studies on healthy young adults involve placement of the MA device at the SN for recordings performed simultaneously with RIP and nasal airflow. The RIP bands mount around the midsternal level (rib cage) and abdomen (Fig. 2a), analogous to lung volume changes during respiration. Nasal pressure measured using a standard nasal cannula connected to a pressure transducer is a surrogate for airflow. The RIP results in Fig. 2c follow from band-pass (0.1–5 Hz) and subsequent band-stop (5–799 Hz) filtering of data collected at 2 kHz. Band-pass filtering (0.1–0.8 Hz) data from the MA device (*z*-axis accelerometry) highlights respiratory cycles. Results of high-pass filtering (90 Hz) reveal features related to swallowing. Swallow events also include a low-frequency signature associated with movements and bolus flow during pharyngeal swallowing. Measurements of resting breaths (Fig. 2d), swallows of 5, 10, 20, and 40 ml of thin liquids, and spontaneous swallows of saliva can be detected using RIP bands and MA device, with an excellent agreement (91%) for subjects while seated without movements. A corresponding Bland–Altman plot (Supplementary Fig. 1) compares the number of detected swallows from the MA device to the RIP bands for 61 young and healthy adults (33 male and 28 female, mean age = 28.3), corresponding to a total of 1050 swallows. Quantitative comparisons of detected swallows indicate that mean differences for counted swallows are −0.31 for this cohort of *n* = 67 subjects. These results demonstrate the feasibility in monitoring breathing and swallowing in a wireless, noninvasive manner, continuously and in nearly any scenario, even during natural daily activities. Such features are unavailable in any other technology^17,35–37^, to the best of our knowledge.

Additional validation studies involve modified Barium swallow studies (MBSS) on patients with dysphagia resulting from chronic obstructive pulmonary disease (COPD), head and neck cancer, and Parkinson’s disease (PD). MBSS allows for real-time fluoroscopic visualization of swallowing movements relative to bolus flow in the upper digestive tract and the penetration/aspiration events^38–40^. Synchronized data from MBSS (Fig. 2b), RIP bands, nasal cannula, and MA recordings on three patients with dysphagia demonstrate swallow-respiratory phase patterns (COPD: Inhalation-IN/IN, Head and Neck Cancer: IN/Exhalation-EX, PD: IN/IN) consistent with impaired respiratory-swallow coordination (Fig. 2e). These preliminary studies demonstrate the therapeutic utility of this device for patients with dysphagia suffering from COPD, PD, and head and neck cancer. Additional studies with the dysphagia population represent topics of current investigation.

### Swallowing motions quantified by 3D digital image correlation (3D-DIC)

**Fig. 3:**
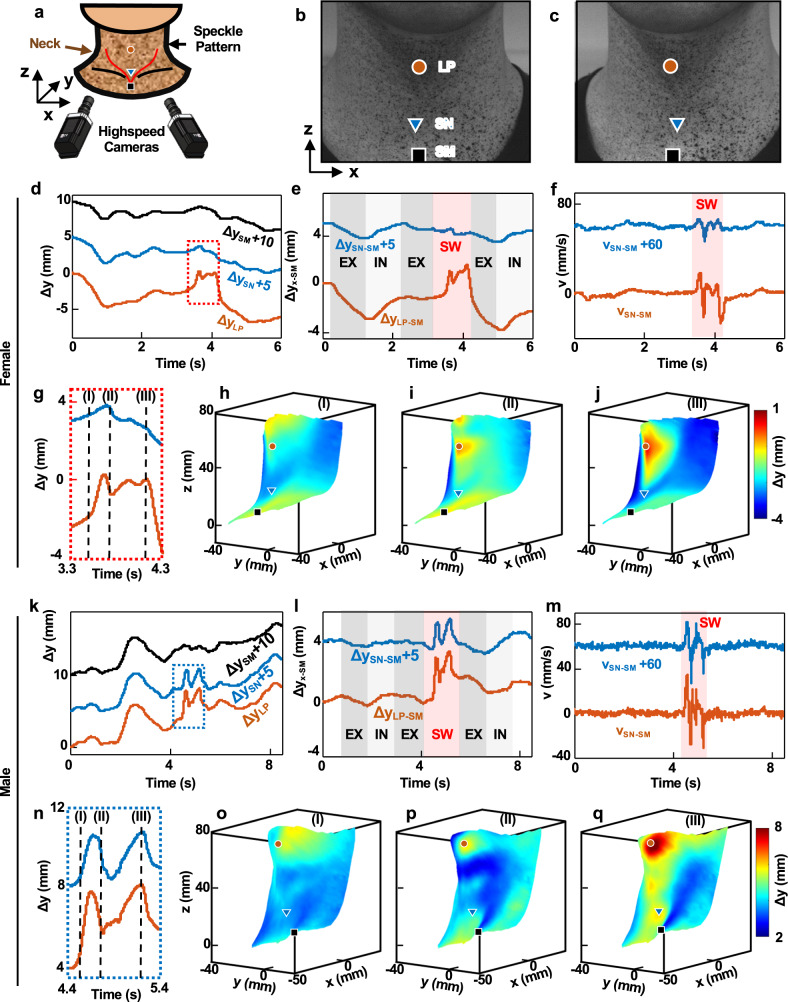
3D-DIC measurements from a female and a male subject before, during, and after a representative swallow event. **a** Experimental setup for 3D-DIC. **b**, **c** Example of a pair of images collected with this system. Red circle, blue triangle, and black square symbols identify the laryngeal prominence (LP), suprasternal notch (SN), and sternal manubrium (SM), respectively. Displacements along the *z*-axis (Δ*z*) function of time at the LP, SN, and SM during breathing and swallowing for the **d** female and **k** male subjects. The curves are offset along the *z*-axis by 5 and 10 mm for ease of viewing. Differential displacements, Δ*z*_LP_–Δ*z*_SM_ (Δ*z*_LP−SM_) and Δ*z*_SN_–Δ*z*_SM_ (Δ*z*_SN−SM_) for the **e** female and **l** Male subjects. The data are offset along the *z*-axis by 5 mm for ease of viewing. Temporal derivative of differential displacements, v, for the **f** female and **m** male subjects. Magnified representation of the *z*-displacements during swallowing during (I) the end of the oral phase, (II) the middle of the pharyngeal phase, and (III) the beginning of the esophageal phase for the **g** female and **n** male subjects. 3D maps of displacements corresponding to (I), (II), and (III) for the **h**–**j**. Female and **o**–**q** male subjects.

Swallowing involves the coordination of rapid and overlapping movements of structures in the oral cavity, pharynx, and esophagus, each of which can vary with age, gender, bolus volume, anatomic features, and other factors^41,42^. A quantitative study of these effects in the spatiotemporal domain, using 3D digital image correlation (3D-DIC), provides insights into features observed in MA data. Figure 3 shows 3D maps of displacements associated with swallowing motions that occur in the middle of exhalation across the neck of healthy female (32 years old) and male (29 years old) subjects, including regions of the LP, SN, and SM (Fig. 3a–c). The distances between the LP and SN of the female and male subjects are 38.4 and 49.4 mm, respectively. Out-of-plane displacement profiles, Δ*z*, of those three points of interest show signatures of swallowing, highlighted by purple boxes. Overall body motions in these examples overshadow respiratory activity in both cases (Fig. 3d, k). Differential displacements at the LP and SM, Δ*z*_LP__−__SM_, and SN and SM, Δ*z*_SN__−__SM_, show respiratory dynamics associated with breathing cycles (Fig. 3e, l). The time variation of Δ*z*_SN__−__SM_ for the female subject does not exhibit a distinct feature associated with swallowing, suggesting that SN is not an appropriate measurement location in this case. The temporal derivative of Δ*z*_SN__−__SM_, v_SN__−__SM_, during swallowing for the female subject is ~70.6% less than v_LP__−__SM_ and ~73.8% less than the v_SN__−__SM_ for the male subject.

Displacement profiles in Fig. 3g, n highlight some crucial details. Of the two distinct local peaks observed at the LP for the female and male subjects, the first likely represents the elevation of the larynx as the bolus passes through the pharynx. The second peak, followed by the concave-up region, potentially indicates the entry of the bolus through the upper esophagus. Representative 3D maps illustrate additional details at the end of the oral phase (Fig. 3h, o), the middle of the pharyngeal phase (Fig. 3i, p), and the beginning of the esophageal phase. The displacement fields do not change significantly throughout the swallowing motion at the SN and its vicinity for the female subject, consistent with the displacement profiles. These results motivate an option to mount one IMU at the LP for subjects with weak swallow motions at the SN. The narrow, thin umbilical connection supports a comfortable interface at this location while allowing the second IMU to be positioned at the SM for differential measurements and tracking of respiratory activity. The 3D imaging results quantitatively capture these variations in displacement profiles and their dependence on body type and physiology. The IMU mounted on the umbilical part of the device allows the device to be positioned on the upper neck in a comfortable manner suitable for a broad range of individuals, including patients with dysphagia. UMA data show trends similar to those illustrated here (Supplementary Fig. 2).

### Differential sensing of swallow and respiratory activity

**Fig. 4:**
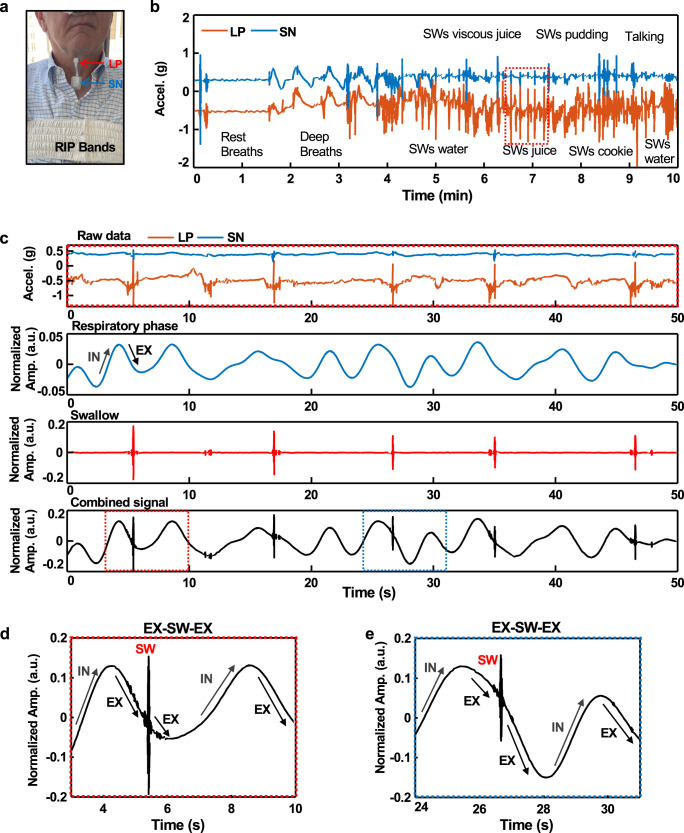
Measurements from a patient with Parkinson’s disease. **a** Image of the subject with an umbilical mechano-acoustic (UMA) device and RIP bands. **b** Raw data from the UMA device, corresponding to accelerations measured at the SN (blue) and LP (orange). The activities illustrated here include three different respirations followed by swallows associated with various liquids and food. **c** Graphs from top to bottom show data: unprocessed, corresponding to swallowing of juice as illustrated in the red dashed box of the frame **a**; bandpass filtered between 0.1 and 0.8 Hz; high pass filtered at 90 Hz; combined, filtered data to highlight swallowing and respiration. **d**, **e** Data corresponding to the regions highlighted by the red and blue dashed boxes in the bottom graph of frame **c**.

Tests on patients who suffer from dysphagia provide insights into the detection of respiratory phases and swallowing events, even during daily activities. Figure 4 shows an example of the device on a patient with PD, positioned such that IMU1/IMU2 are on the LP and SN, respectively. Supplementary Fig. 6 shows the correlation between data from the UMA device and RIP bands for different types of breathing. The results indicate consistent respiratory trends during sedentary breathing and swallowing. Figure 4b presents unprocessed UMA data for various kinds of breathing behaviors and swallowing different food and liquid viscosities. Figure 4c shows data from the highlighted area in Fig. 4b, with periods of IN and EX identified using data from IMU2 after band-pass filtering (0.1–0.8 Hz). High-pass filtering (90 Hz) of the data from IMU1 highlights swallowing events. The combined signals reveal the timing of swallows relative to the respiratory phase. Figure 4d, e plots this combined signal (respiration + swallow) over a time interval during which a swallowing event occurs during the EX-phase.

**Fig. 5:**
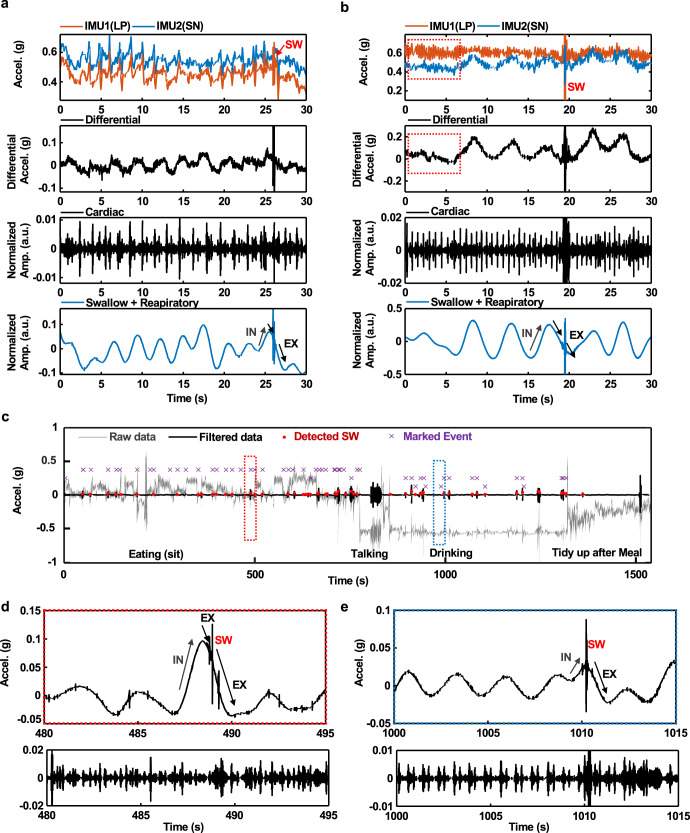
Differential sensing for measurements of respiratory cycles and swallowing events during physical activities. **a** MA data from the LP and SN while walking. Graphs from top to bottom show raw MA signals, differential MA results with band-pass filtering from 90 to 200 Hz to highlight swallowing, differential MA results with band-pass filtering from 20 to 50 Hz to highlight cardiac cycles, and differential MA results with low-pass filtering at 0.8 Hz and band-pass filtering from 90 to 200 Hz to highlight combined events of swallowing and respiratory activity. **b** Results correspond to those in Fig. 5a but for the case of simulated tremors with frequencies between 4 and 5 Hz. **c** Detection of swallowing events during natural daily activities. Swallows were detected while eating dinner, seated at a table, and drinking water during walking, with 89.6% sensitivity and 87.8% precision. The data include confounding activities such as talking and organizing tableware after eating. **d**, **e** Data corresponding to the regions highlighted by the red and blue dashed boxes in **c**. The bottom frames show data after band-pass filtering from 20 to 50 Hz to highlight cardiac activity.

As described previously, differential signals from these two IMUs reduce motion artifacts to enable accurate tracking of swallows and respirations during routine daily activities. Figure 5a shows differential data captured from a patient with PD while walking to reveal clear respiratory, swallowing, and cardiac activity signatures with minimal confounding features associated with body motions. Another example is capturing these features during pill-rolling tremors (4–6 Hz), as observed in 46~93.4% of patients with PD^43–45^. An example of measurement of swallowing events in this scenario is in Fig. 5b and Supplementary Fig. 5. As in other cases, the cycles of respiration also appear prominently. Results for measurements during other movements, such as swinging back and forth, walking, running, and cycling, are seen in Supplementary Fig. 6. Figure 5c shows UMA device output while attached to a subject during typical evening post-dinner activities. After eating, the subject engaged in conversations, drank tea and moved around clearing dishes after the meal for ~25 min. Results indicate detection of swallow events while eating, drinking, and intermittent un-cued saliva swallowing with 89.6% sensitivity and 87.8% precision. As in Fig. 5d, e, the data also reveal the timing of swallowing events relative to the respiratory phase.

### Real-time analytics for visual and haptic feedback

**Fig. 6:**
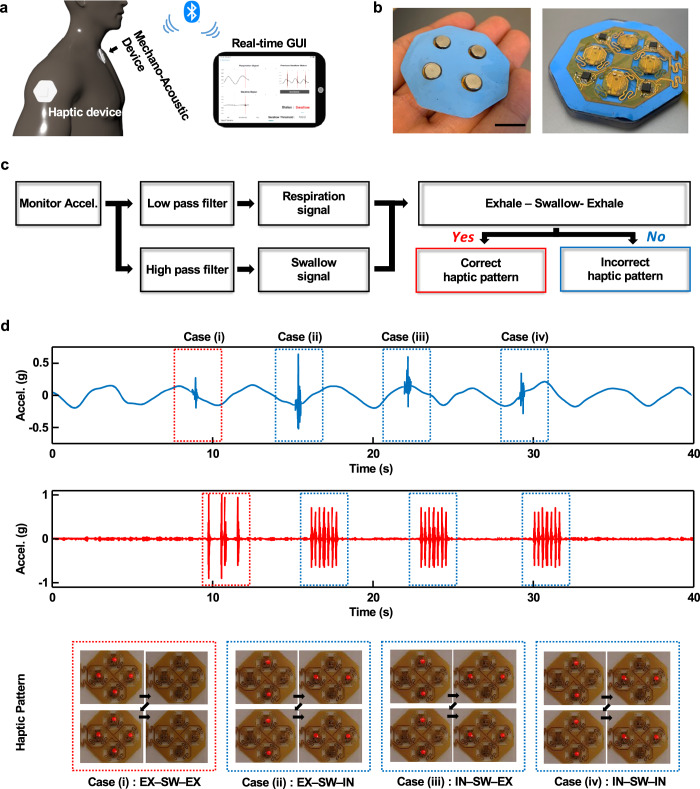
Real-time swallow detection and haptic feedback. **a** Schematic illustration of real-time swallow detection and haptic feedback using BLE communication. **b** Photograph of a wireless haptic feedback system that includes four brush type eccentric rotating mass (ERM) vibration motors. **c** Block diagram and haptic feedback mechanisms for real-time swallow detection system (scale bar = 2 cm). **d** Swallow and respiratory signals of four swallow-respiration cases: (i) EX/SW/EX, (ii) EX/SW/IN, (iii) IN/SW/EX, and (iv) IN/SW/IN) (upper graph). Acceleration results from haptic vibration patterns according to the respiratory phase with swallow initiation (lower graph). Photographs show the haptic patterns of vibration via red LED indicators.

Real-time visual and haptic feedback provides additional value for the rehabilitation of patients suffering from dysphagia, as an example of the clinical utility of the device and associated detection algorithms. The BLE 5.0 protocol allows for fast data transmission to host electronic devices, such as smartphones or tablets. Real-time data analysis using algorithms described previously and graphical representation of the results can be achieved using the computational capabilities of these devices, thereby expanding the potential applications to continuous feedback and training. The schematic illustration and image in Fig. 6a, b, respectively, highlight such a system, along with an optional skin-interfaced haptic device (4 × 4 cm) that incorporates four brush-type eccentric rotating mass (ERM) vibration motors and a BLE interface to the MA device, for user feedback. A digital pulse width modulated (PWM) signal with amplitude shift key modulation sets the vibration power of each of these actuators independently and in a fashion coordinated with the results of analysis of data from the UMA to yield various spatio-temporal haptic patterns according to swallowing events and respiratory phase before and after, as seen in Fig. 6c. An example appears in the upper graph of Fig. 6d for case (i), corresponding to EX-SW-EX, the optimal respiratory-swallow phase pattern that is observed in healthy adults. The corresponding haptic pattern involves all four actuators operated three times in synchrony at 500 ms intervals. As in cases (ii)–(iv), suboptimal respiratory-swallow phase pattern activates the vertical actuators, and the side actuators sequentially vibrate at 250 ms intervals. Vibrations detected using an accelerometer, and a red light-emitting diode (LED) illuminated within 800 ms of swallowing appear in the bottom graph and images in Fig. 6d. On-going work focuses on the clinical implementation of this haptic module for therapy and training.

This paper summarizes a sensor and a corresponding set of analysis algorithms specially designed to support simultaneous, real-time monitoring of swallowing and breathing behaviors for patients with dysphagia. Specifically, the device monitors movements captured from the surface of the skin at two distinct locations on the neck and adjacent regions (SN/SM or SN/LP) to capture both swallow and respiratory phase signatures, applicable across a wide range of individuals, including patients with dysphagia. A differential sensing mode allows for reliable operation even during natural daily activities. A real-time graphical user interface and an optional haptic module support visual and haptic feedback, respectively. Benchtop and pilot studies on healthy subjects and patients with PD highlight key features of the devices and their potential for broad utility in clinical research and at-home settings to guide the treatment of patients with dysphagia. Simple algorithms for identifying swallowing events and corresponding respiratory phase offer excellent performance with modest computational load, thereby allowing for reliable, real-time analysis on portable electronic devices. Developing advanced approaches based on machine learning represents an area of current work. This technology platform has strong potential for the treatment and care of patients suffering from dysphagia, directly through its use in improved training protocols and indirectly through its use in studies of swallow dynamics.

## Methods

### Fabrication and assembly

Electronic design automation software (EAGLE ver. 9.6.1, AUTODESK) yielded circuit schematics and designs for fPCBs produced by an outside vendor (PCBWay, Inc). Rinsing the fPCBs in isopropanol (Fisher Scientific) removed contaminants and surface oxides, preparing them for chip and component assembly. A non-conductive epoxy (Loctite 3621, Henkel) mechanically bonded surface mount components (power management integrated circuit, IMUs, BLE SoC, memory, regulators, and all passive components) to the fPCB. Reflow soldering (Weller WTHAIN, Weller Tools) with low-temperature solder paste (TS 391LT, ChipQuik) established electrical contacts.

The UMA devices included the following elements: (1) power management electronics (BQ2510A, Texas Instruments) and a rechargeable Li-Po battery (75 mAh) to supply power to the system, (2) NFC charging components and rectifiers to enable wireless charging, (3) dual IMUs (LSM6DSL, STMicroelectronics) to acquire MA signals, (4) 8 Gb NAND flash memory (MT29F8G08, Micron Technology) to store acquired data, (5) BLE SoC (5.0) microcontroller (nRF52840, Nordic Semiconductor) with a Bluetooth radio to enable wireless data collection, and (6) associated passive components. General-purpose input/output (GPI/O) pins on the BLE SoC defined interfaces to the dual IMUs, NAND flash, and power management components via SPI.

The haptic devices included the following elements: (1) a rechargeable Li-Po battery (45 mAh) to supply power to the system, (2) NFC charging components and rectifiers to enable wireless charging, (3) BLE SoC (5.1) microcontroller (CC2640, Texas Instruments) and antenna chip (2450AT18D0100, Johanson Tech.) with a Bluetooth radio to enable wireless data collection, and (4) four brush-type ERM (CLP0820B004L, Jinlong Machinery & Electronics) actuators connected to the four GPI/O pins of the microcontroller (ATMega328P, Microchip Technology) powered using a PWM signal to create a haptic sensation. The fPCB for this system incorporates a serpentine structure to minimize crosstalk between actuators and enhance mechanical flexibility.

### Soft encapsulation

Tri-axis CNC milling (Modela MDX-540, Roland) formed female and male aluminum molds. Spacers between these molds created a gap of 0.3 mm to define the thickness of the molded silicone shell. Casting medical-grade thermoset silicone elastomer (prepolymer and curing agent) into the female mold (Silbione-4420, Elkem) and aligning and compressing the male mold to a force of 8000 N with a hot press (AutoFour 3012, Carver) for 10 min at 90 °C formed a shell. Separately, spin casting a silicone elastomer (Silbione-4420, Elkem) on a clean (poly)methyl methacrylate plate (200 rpm for 60 s) and thermally curing (70 °C for 25 min) defined a thin film for the bottom layer of the device. Mounting the fPCB onto the bottom layer and pouring uncured liquid prepolymer of silicone along the outline (Ecoflex 00-30, Smooth-On Inc.) on the top layer and curing (70 °C for 30 min) established a mechanical bond to seal the fPCB from the external environment. Lastly, a CO_2_ laser cutting system (VLS2.30, Universal Laser Systems) formed the final outline of the device. The same system also defined corresponding outlines for a medical-grade, dual-sided silicone-acrylate adhesive (2477p, 3M) designed to bond the device to the skin. A similar process defined the encapsulation for the wireless haptic feedback device, with cutouts for the ERM actuators.

### Validation study experiments

RIP, nasal airflow, and MA signals were acquired synchronously during the MBSS. The wearable sensor was affixed to the SN. The signals were coupled with the TIMS OEM Medical System for real-time, time-synced data acquisition during MBSS. At the beginning of the MBSS, with fluoroscopy off, each participant was instructed to complete 2–3 min of rest breathing until stable, quiet breathing was established and recorded. The Modified Barium Swallow Impairment Profile (MBSImP), a standardized, reliable, and validated approach for administration, interpretation, and quantification of the MBSS, was used in this study^38,46^. The MBSImP protocol includes 12 swallowing tasks of standardized viscosities and volumes in lateral (10 tasks) and AP (2 tasks) views. All trials were self-administered by the participant instructed to “Swallow in your natural way when you are ready.” No cues were given about when to initiate a swallow to avoid unintentional influence of cueing on respiratory-swallow phase patterning.

### 3D digital image correlation experiments

Swallowing motions of healthy female (32 years old) and male (29 years old) subjects captured by 3D-DIC during inhalation, exhalation, and swallows revealed the kinematics of the process. The measurement used two high-speed 2 megapixel cameras operating at a frame rate of 500 fps, subsequent image processing with the open-source 3D-DIC software, MultiDIC^47^. The investigation area was 120 × 60 × 90 mm^3^, covering the entire neck of both subjects. The DIC subset radius and spacing were 20 and 10 pixels, resolving over 3500 grids. Supplementary Fig. 2 shows correlations between displacements from the digital image and accelerometry results from the MA device. Supplementary Fig 2a presents Δ*z*_LP−SM_ results from Fig. 3l. Supplementary Fig. 2b features low and high pass filtered results to highlight the respiratory phase and swallowing. Yellow, purple, and green shaded regions correspond to inhalation (IN), exhalation (EX), and swallowing (SW), respectively. Supplementary Fig. 2c presents MA signals that correspond to intervals in Supplementary Fig. 2a, b. The trends in these results are consistent for breathing and swallowing. Figure 3 illustrates movements of the chest wall and the corresponding change in the orientation of the gravity vector relative to the device during inhalation. As the chest volume increases, the sternum rises upwards. Digital images estimate respiration based on the position (*y*) of the ascending sternum—the IMUs at the LP and SM estimate respiration based on changing accelerations. The differential acceleration between these two IMUs is approximately related to *g*sin*θ*, where *θ* is the angle between LP and SM. Here, the + direction of the coordinate *z*-axis is the direction away from the body. On inhalation, *g*sin*θ* increases and thus also the differential acceleration. The opposite occurs during exhalation.

### Differential signal detection

The UMA device has two accelerometers; one is located on LP to detect the swallows of a subject with a weak swallowing signal, and the other is at 7 cm for detecting the respiratory phase. The vibrations acquired at these two positions were aligned with the axis outward of the body using a rotational matrix calculated with values on a plane parallel to the attached body surface. Skin vibrations associated with swallowing, respiration, and cardiac activity had different values at two positions. Accelerations at other locations aligned with the same axis were different for swallowing, respiration, and skin vibrations associated with the heart. In contrast, two accelerometers at two different positions produced an almost identical response to the overall movement of the global body movement. Therefore, by obtaining the difference in acceleration at two positions, it was possible to capture swallowing, respiration, and cardiac-related signal among daily activities.

### Data analytics

Low pass filtering (<0.8 Hz) and bandpass filtering (90–200 Hz) of differential signals yielded information on respiratory cycles and swallowing, respectively. The amplitude of the former was generally smaller than that of the latter. To aid in graphical visualization, the respiratory signals were multiplied by a factor of four before combining them with the swallow signals (the fourth plot in Fig. 4c–e).

### Swallow detection

A peak detection algorithm with an adaptive threshold identified swallowing events from the swallowing signals. The adaptive threshold value is followed by averaging the signal across a temporal window of 5 s. A swallow corresponded to a signal amplitude that exceeded the threshold. However, any signal with a magnitude larger than 0.07 g was associated with a non-swallowing event such as a cough or a physical impact, as shown in Supplementary Fig. 6a. This adaptive threshold scheme effectively accommodated changes in the behavior of a given subject and differences between subjects, as confirmed in the experimental results of Supplementary Fig. 6b.

### Protocols for human subject studies

The studies were approved by the Northwestern University Institutional Review Board, Chicago, IL, USA (STU00211532 for healthy validation studies, STU00207857 for COPD and Head and Neck Cancer MBSS validation studies, STU00212981 for PD validation studies, and STU00212981 for UMA device validation studies). All study-related procedures were carried out by the standards listed in the Declaration of Helsinki, 1964. For all studies, double-sided silicone acrylate adhesive (3M,2477p) secured the sensor to the neck area. The silicone side of the adhesive supports a gentle interface to the skin, with the ability to support wear times of up to 12 h without risks of delamination. The acrylate side of the adhesive establishes a strong bond to the device. After each data collection session, the device was sterilized with 70% isopropyl alcohol and left to dry at room temperature, and this sterilization process was repeated twice. All participants provided written, informed consent to take part in the study. All participants also consented to the capture and publication of photographs for these studied.

### Reporting summary

Further information on research design is available in the Nature Research Reporting Summary linked to this article.

## Supplementary information

Supplemental Materials

Reporting Summary

## Data Availability

All data needed to evaluate the conclusions are present in the paper and/or in the Supplementary Materials. Additional data and materials may be requested from one of the corresponding authors.
