# Supplementary material for: Soft skin-interfaced mechano-acoustic sensors for real-time monitoring and patient feedback on respiratory and swallowing biomechanics
Source: NPJ Digit Med. 2022 Sep 20;5:147. doi: 10.1038/s41746-022-00691-w (PMC9485153; doi:10.1038/s41746-022-00691-w)
Supplement: Supplementary file 1 — Supplemental Materials [file 41746_2022_691_MOESM1_ESM.pdf]

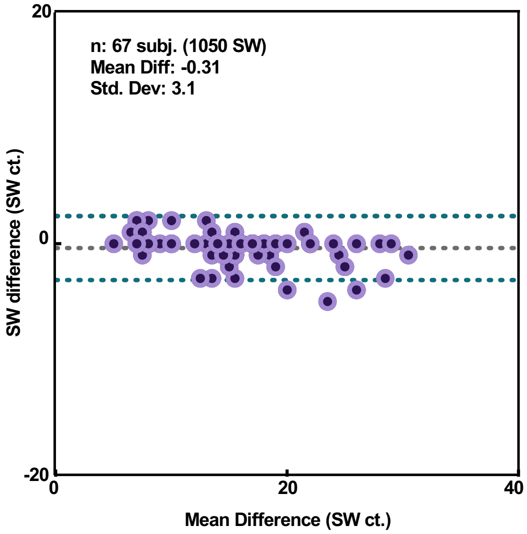

Supplementary Figure 1. Correlation between MA device and RIP band for respiratory phase from patient data. A Bland Altman plot comparing the number of detected swallows captured with the RIP bands and nasal cannula (Inductotrace system) to the MA device were compared to detected swallows captured with the MA sensor with excellent agreement (91%) for subjects while seated without movements. (n=61 subjects, 1050 total swallows).

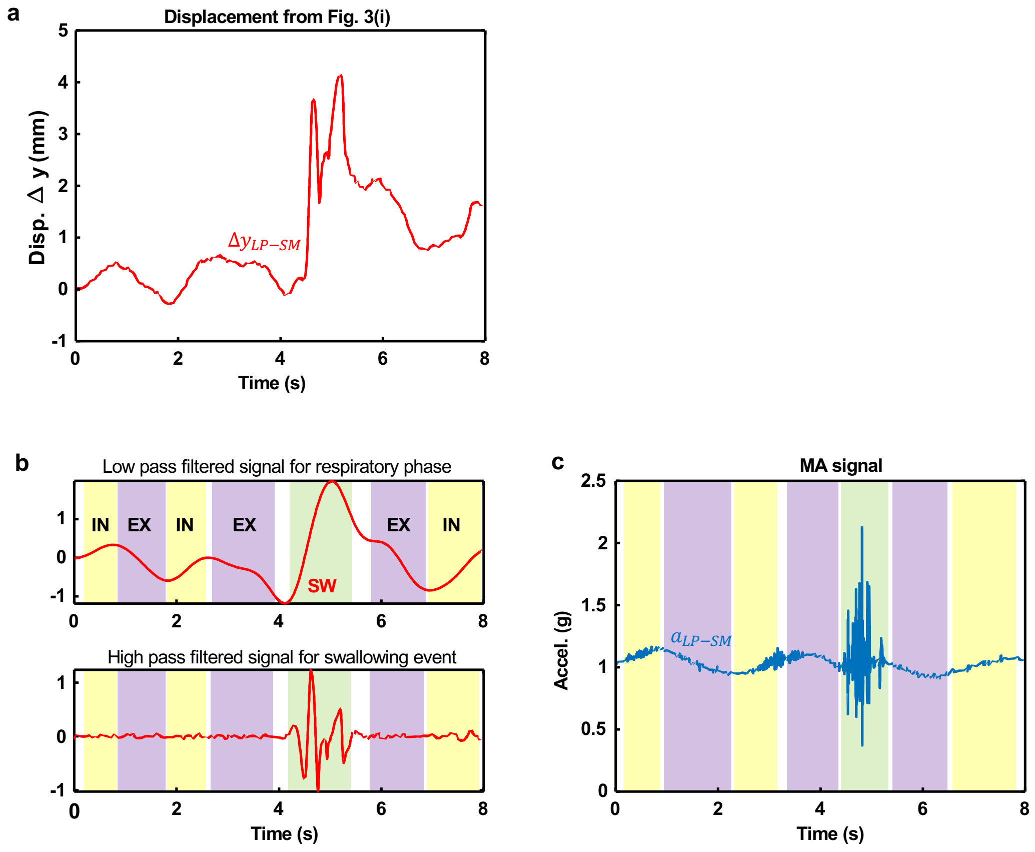

Supplementary Figure 2. Correlation between displacement from camera images and from the MA device. **a.** Differential displacement between the LP and SN from Fig. 2(i). **b.** Respiratory phase and swallowing indication in low and high-pass filtered signals obtained using differential displacement data from Fig S2a. Yellow shaded regions indicated by IN correspond to inhalation, purple shaded regions indicated by EX correspond to exhalation, and the green shaded region indicated by SW corresponds to a swallowing event. **c.** Corresponding differential signal from a MA device with IMUs on the LP and SM.

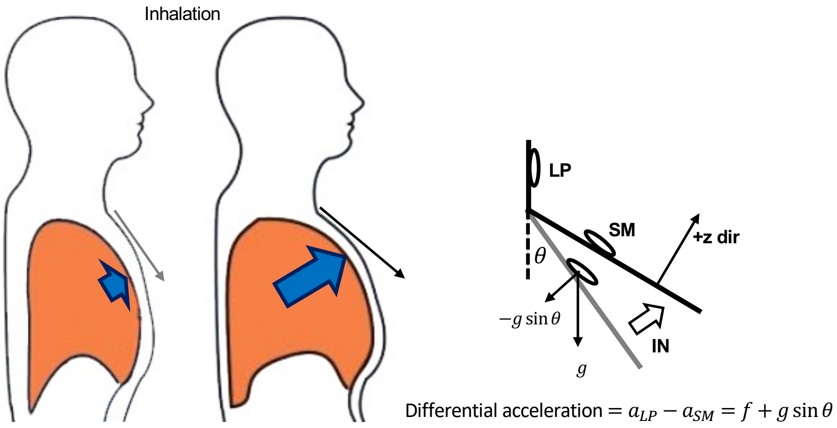

Inhalation: Sternum moves upward → θ ↑ → g sinθ ↑ → Diff. accel. from MA device ↑
Exhalation: Sternum moves downward → θ ↓ → g sinθ ↓ → Diff. accel. from MA device ↓

Supplementary Figure 3. Illustration of the chest movement and the acceleration change while inhalation. The differential acceleration defined as a numerical value related to $g\sin\theta$ due to the gravitational acceleration g. On inhalation, theta (θ), the angle between the neck and the chest bone, increases, which increases $g\sin\theta$ and increases the differential acceleration measured in the MA device. Conversely, on exhalation, θ decreases and the differential acceleration decreases. The differential signal from the MA device and the differential displacement from the camera image have the same trend for respiration.

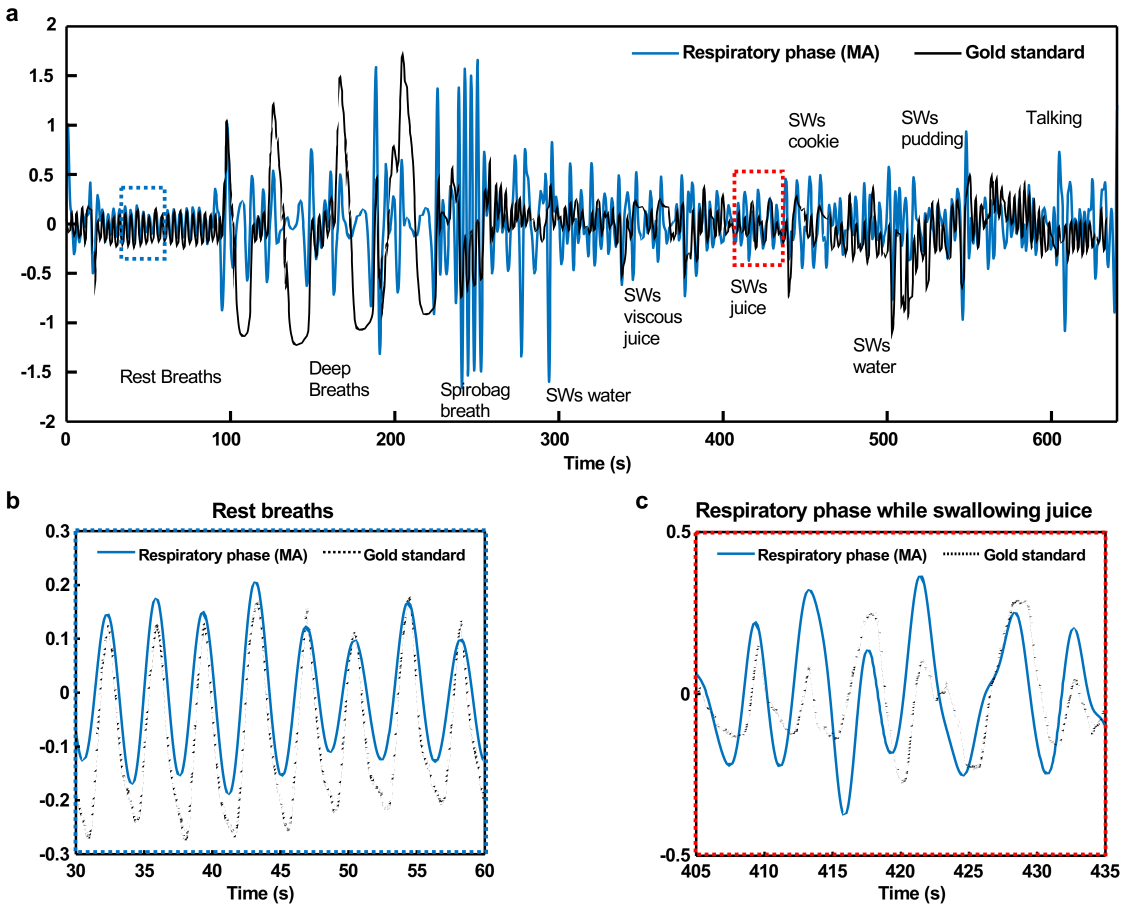

Supplementary Figure 4. Correlation between data from a MA device and RIP bands for respiratory phase collected from patients. a. Blue line indicates low pass filtered signal from Fig. 4b and black dashed line indicates respiratory phase from RIP bands. **b.** Respiratory phase when the subject inhales and exhales normally. Data associated with respiratory phase from the MA device and RIP bands across a time interval highlighted by the blue box in Fig. S4a. **c.** Respiratory phase when the subject swallowed juice. Data from the time interval highlighted by the red box in Fig. S4a.

a **b**

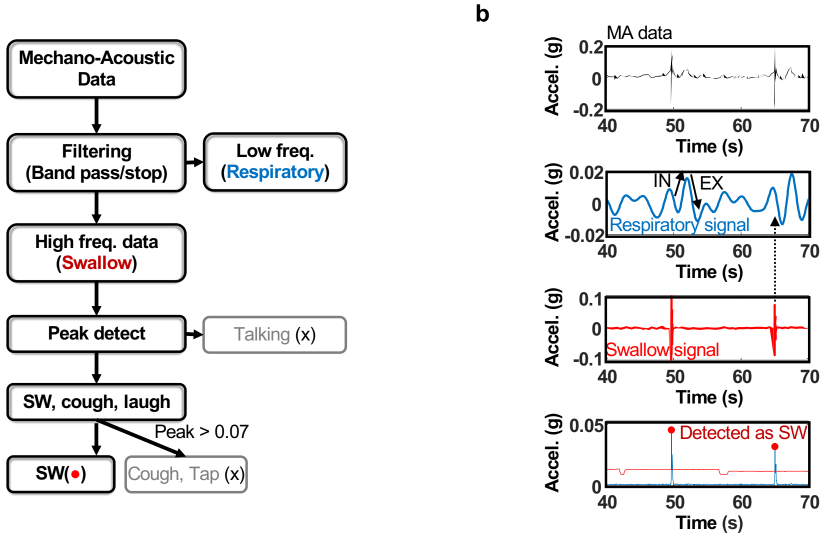

c

	Subjects
Number of subjects	8
Age	22 – 68 yr
Gender Ratio (M:F)	1 : 1
Avg. Trial Swallows	65 times
Type of Swallow	7
Avg. Non-Swallow Time	25 min

d

Single	S	B&F	W	C	R
**Accuracy**	87.1	84.4	84.8	80.4	78.2
**Precision**	70.6	66.7	67	60.6	63.2
**Sensitivity**	86.7	79	83.8	70.4	61.4
**Specificity**	87.3	86.3	85.2	84	85.2

e

Dual	S	B&F	W	C	R
**Accuracy**	89.3	88.3	88.4	82.3	81.2
**Precision**	74.3	73.9	72.3	63.8	67.6
**Sensitivity**	90.4	84	91.3	84.4	68.6
**Specificity**	89	89.7	87.4	85.2	86.4

S: Sitting, B&F: Swaying back and force
W: Walking, C: Cycling, R: Running

Supplementary Figure 5. Swallow detection algorithm and results. a. Flowchart of the algorithm to detect swallowing events and representative signal for each step. **B.** From raw data MA data, low pass filtering yields the respiratory phase, and high pass filtering with peak detection defines swallowing event. **c.** Information associated with a healthy normal subject for validating the algorithm. **d.** Detection results based on data from IMU 1 mounted on the SN for swallowing during various activities including sitting, swaying back and force, walking, cycling and running. **e.** Detection results based on differential accelerometer data for swallow during these same activities.

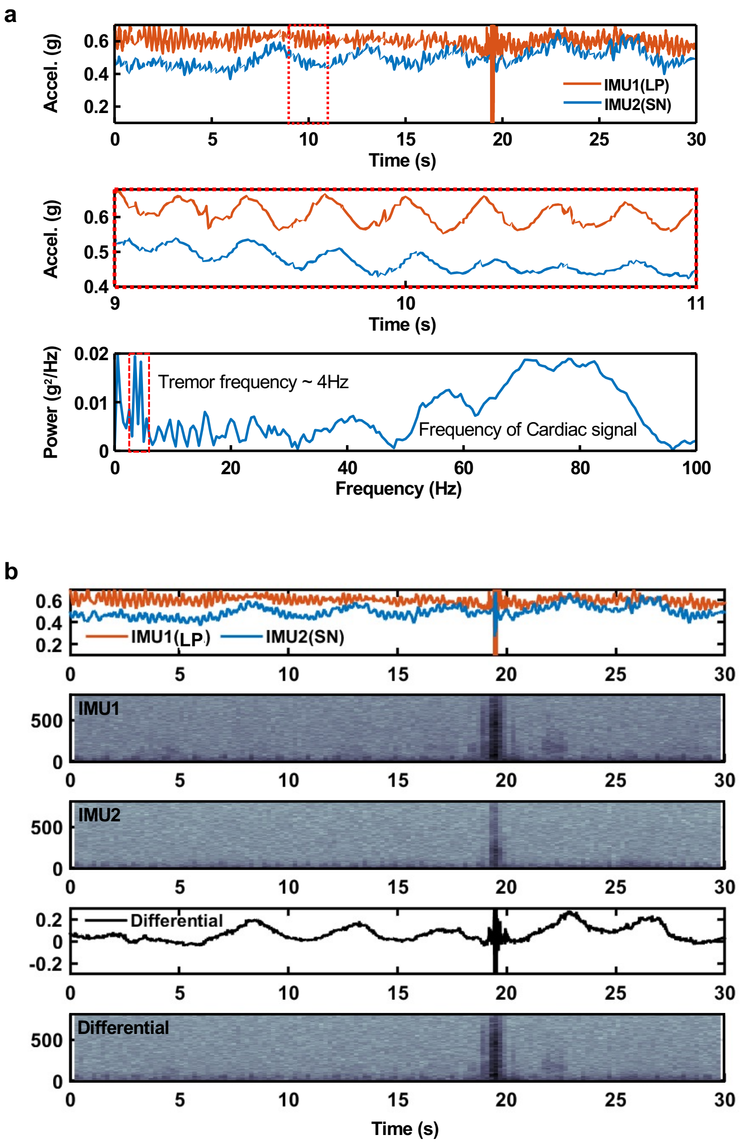

Supplementary Figure 6. Frequency analysis of action tremor. a. Unprocessed data from Fig. 5b, magnified view of tremor action for 2 s, and power spectrum. The dominant frequency due to the tremor action is around 4 Hz, consistent with the range of 4~6 Hz for pill-rolling tremors. **b.** Unprocessed data and spectrogram results from IMU1 and IMU2. Differential accelerometer data and its spectrogram.
