# Supplementary material for: Soft skin-interfaced mechano-acoustic sensors for real-time monitoring and patient feedback on respiratory and swallowing biomechanics
Source: NPJ Digit Med. 2022 Sep 20;5:147. doi: 10.1038/s41746-022-00691-w (PMC9485153; doi:10.1038/s41746-022-00691-w)
Supplement: Supplementary file 2 — Reporting Summary [file 41746_2022_691_MOESM2_ESM.pdf]

nature portfolio

Corresponding author(s): Bonnie Martin-Harris, John A. Rogers

Last updated by author(s): Aug 9, 2022

Reporting Summary

Nature Portfolio wishes to improve the reproducibility of the work that we publish. This form provides structure for consistency and transparency in reporting. For further information on Nature Portfolio policies, see our Editorial Policies and the Editorial Policy Checklist.

Statistics

For all statistical analyses, confirm that the following items are present in the figure legend, table legend, main text, or Methods section.

n/a	Confirmed	
☐	☒	The exact sample size (n) for each experimental group/condition, given as a discrete number and unit of measurement
☐	☒	A statement on whether measurements were taken from distinct samples or whether the same sample was measured repeatedly
☐	☒	The statistical test(s) used AND whether they are one- or two-sided<br>*Only common tests should be described solely by name; describe more complex techniques in the Methods section.*
☒	☐	A description of all covariates tested
☒	☐	A description of any assumptions or corrections, such as tests of normality and adjustment for multiple comparisons
☐	☒	A full description of the statistical parameters including central tendency (e.g. means) or other basic estimates (e.g. regression coefficient) AND variation (e.g. standard deviation) or associated estimates of uncertainty (e.g. confidence intervals)
☐	☒	For null hypothesis testing, the test statistic (e.g. F, t, r) with confidence intervals, effect sizes, degrees of freedom and P value noted<br>*Give P values as exact values whenever suitable.*
☒	☐	For Bayesian analysis, information on the choice of priors and Markov chain Monte Carlo settings
☒	☐	For hierarchical and complex designs, identification of the appropriate level for tests and full reporting of outcomes
☒	☐	Estimates of effect sizes (e.g. Cohen's d, Pearson's r), indicating how they were calculated

Our web collection on statistics for biologists contains articles on many of the points above.

Software and code

Policy information about availability of computer code

Data collection	All software analysis was performed on MATLAB and is available on reasonable request from one of the senior authors of the study.
Data analysis	All algorithms used for processing and displaying data are described in the text.

For manuscripts utilizing custom algorithms or software that are central to the research but not yet described in published literature, software must be made available to editors and reviewers. We strongly encourage code deposition in a community repository (e.g. GitHub). See the Nature Portfolio guidelines for submitting code & software for further information.

Data

Policy information about availability of data

All manuscripts must include a data availability statement. This statement should provide the following information, where applicable:

- Accession codes, unique identifiers, or web links for publicly available datasets
- A description of any restrictions on data availability
- For clinical datasets or third party data, please ensure that the statement adheres to our policy

All data needed to evaluate the conclusions are present in the paper and/or in the Supplementary Materials. Additional data information and materials may be requested from one of the corresponding authors.

Human research participants

Policy information about studies involving human research participants and Sex and Gender in Research.

Reporting on sex and gender	Sex and gender were not considered in the study design,and the recruited population characteristics were close to evenly split across males and females. Sex and/or gender data was collected from patients self reporting this data. There was no gender/sex based analysis performed because swallow/respiratory phase coordination is a common issue affecting all sexes and genders.
Population characteristics	Population characteristics for healthy subjects: n=61 (Age range: 20-42), 33 male and 28 female, mean age=28.3
Recruitment	Patients were recruited from existing patient populations at Northwestern Memorial Hospital in Chicago, IL.
Ethics oversight	Northwestern University

Note that full information on the approval of the study protocol must also be provided in the manuscript.

Field-specific reporting

Please select the one below that is the best fit for your research. If you are not sure, read the appropriate sections before making your selection.

☒ Life sciences ☐ Behavioural & social sciences ☐ Ecological, evolutionary & environmental sciences

For a reference copy of the document with all sections, see nature.com/documents/nr-reporting-summary-flat.pdf

Life sciences study design

All studies must disclose on these points even when the disclosure is negative.

Sample size	This work represents an early stage pilot study and as such, sample size calculations were not performed. The sample size (n=67) was determined by patient availability.
Data exclusions	No data were excluded from any of the analyses.
Replication	All in vitro data was replicated (multiple swallows were detected during long data collection sessions). On body measurement trends (swallow/respiratory phase coordination) were measured across multiple healthy subjects as well as patients suffering from dysphagia.
Randomization	The focus of this study was to demonstrate the clinical feasibility of this device to detect respiratory phase and swallow coordination, and as such randomization was not relevant to this study.
Blinding	Investigators were not formally blinded to the results

Reporting for specific materials, systems and methods

We require information from authors about some types of materials, experimental systems and methods used in many studies. Here, indicate whether each material, system or method listed is relevant to your study. If you are not sure if a list item applies to your research, read the appropriate section before selecting a response.

Materials & experimental systems

n/a	Involved in the study	
☒	☐	Antibodies
☒	☐	Eukaryotic cell lines
☒	☐	Palaeontology and archaeology
☒	☐	Animals and other organisms
☒	☐	Clinical data
☒	☐	Dual use research of concern

Methods

n/a	Involved in the study	
☒	☐	ChIP-seq
☒	☐	Flow cytometry
☒	☐	MRI-based neuroimaging
